# Tonsillectomy as prevention and treatment of sleep-disordered breathing: a report of 23 cases

**DOI:** 10.1186/s40902-016-0092-y

**Published:** 2016-11-25

**Authors:** Jae-Man Woo, Jin-Young Choi

**Affiliations:** 1 Department of Oral and Maxillofacial Surgery, Seoul National University Dental Hospital, 101 Daehakno, Jongno-Gu, Seoul, 110-768 Republic of Korea; 2 Department of Oral and Maxillofacial Surgery, School of Dentistry, Seoul National University, Seoul, Republic of Korea

**Keywords:** Tonsillectomy, Sleep-disordered breathing, Obstructive sleep apnea, Mandibular setback

## Abstract

**Background:**

The paradigm of tonsillectomy has shifted from a treatment of recurrent throat infection to one of multi-discipline management modalities of sleep-disordered breathing (SDB). While tonsillectomy as a treatment for throat problems has been performed almost exclusively by otorhinolaryngologists, tonsillectomy as a part of the armamentarium for the multifactorial, multidisciplinary therapy of sleep-disordered breathing needs a new introduction to those involved in treating SDB patients. This study has its purpose in sharing a series of tonsillectomies performed at the Seoul National University Dental Hospital for the treatment and prevention of SDB in adult patients.

**Methods:**

Total of 78 patients underwent tonsillectomy at the Seoul National University Dental Hospital from 1996 to 2015, and 23 of them who were operated by a single surgeon (Prof. Jin-Young Choi) were included in the study. Through retrospective chart review, the purpose of tonsillectomy, concomitant procedures, grade of tonsillar hypertrophy, surgical outcome, and complications were evaluated.

**Results:**

Twenty-one patients diagnosed with SDB received multiple surgical procedures (uvulopalatal flap, uvulopalatopharyngoplasty, genioglossus advancement genioplasty, tongue base reduction, etc.) along with tonsillectomy. Two patients received mandibular setback orthognathic surgery with concomitant tonsillectomy in anticipation of postoperative airway compromise. All patients showed improvement in symptoms such as snoring and apneic events during sleep.

**Conclusions:**

When only throat infections were considered, tonsillectomy was a procedure rather unfamiliar to oral and maxillofacial surgeons. With a shift of primary indication from recurrent throat infections to SDB and emerging technological and procedural breakthroughs, simpler and safer tonsillectomy has become a major tool in the multidisciplinary treatment modality for SDB.

## Background

Tonsillectomy is one of the most commonly performed surgeries in the head and neck region especially in the pediatric population. Tonsillectomy, by definition, is the complete removal of the palatine tonsils including the surrounding capsules through various surgical methods. Traditionally, tonsillectomies had been performed in children with recurrent throat infections. However, with amassing evidence on the self-limiting characteristic of the hypertrophic tonsils and lack of solid evidence on the efficacy of tonsillectomy in the prevention of recurrent throat infections, the number of surgeries had gradually decreased from the 1970s into the late 1980s [1]. On the other hand, more and more studies have shown that tonsillectomies performed on properly selected pediatric sleep-disordered breathing (SDB) patients can dramatically improve the patients’ breathing. Improvement in breathing has been shown to result in better school performance, physical growth, and general quality of life (QOL). According to a survey study in the USA, there had been a decrease of more than 50 % in tonsillectomy rates from 1977 to 1989. During a similar period of time, the rate of tonsillectomies as the treatment for SDB had greatly increased [1]. Currently, the two major indications for tonsillectomy are recurrent throat infections and SDB, with the latter being the primary indication [1–3].

Along with the primary indication shifting from recurrent throat infections to SDB, tonsillectomy has become a part of a multi-disciplinary treatment armamentarium of SDB therapies. This report has its purpose in sharing accumulated data and experience on tonsillectomies performed in the Seoul National University Dental Hospital, Department of Oral and Maxillofacial Surgery, by a single surgeon from 2006 to 2015. Since SDB including obstructive sleep apnea syndrome (OSAS) is becoming a major topic in the field of oral and maxillofacial surgery, it is important to acknowledge the relationship between hypertrophic tonsils and SDB and to include tonsillectomy as the primary or adjunctive therapy in treating SDB patients.

## Methods

From March 2006 to September 2015, a total of 78 patients received tonsillectomies from six surgeons at the Seoul National University Dental Hospital. Of the 78 patients, 23 of them operated by a single surgeon (Prof. Jin-Young Choi) were included in this study. Patient demographics revealed strong male predilection (male-to-female ratio = 21:2). Mean and median ages of patients were 34.1 and 30 years, respectively. Age of patients ranged from 8 to 63. The purpose of tonsillectomy, concomitant procedures, grade of tonsillar hypertrophy, surgical outcome, and complications were evaluated by way of retrospective chart review. The details of the surgical procedure are laid out in the following section.

### Surgical procedure

**Fig. 1:**
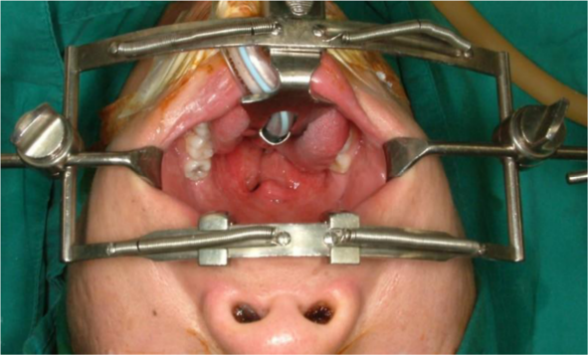
Application of the Dingman retractor

**Fig. 2:**
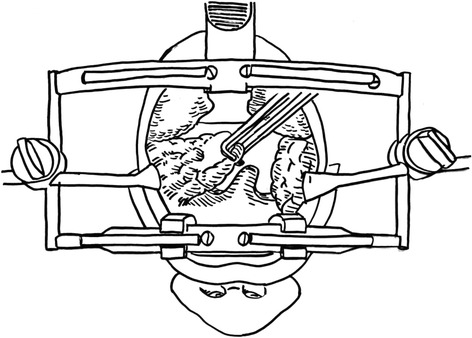
View from the 12-o’clock position after the Dingman retractor application. Traction of medial aspect of Lt. palatine tonsil with right-angled Kelly clamps

**Fig. 3:**
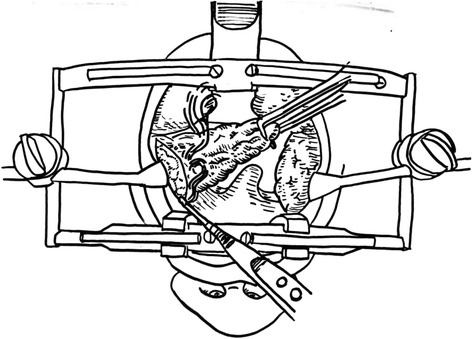
Incision at the base of tonsil with monopolar electrocautery while applying medial traction for tension

**Fig. 4:**
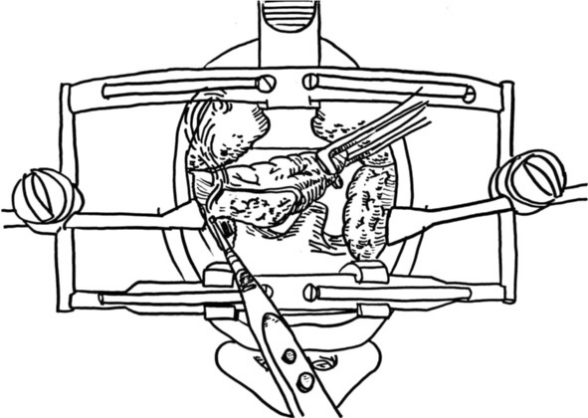
Dissection along extracapsular plane with monopolar electrocautery while applying medial traction

**Fig. 5:**
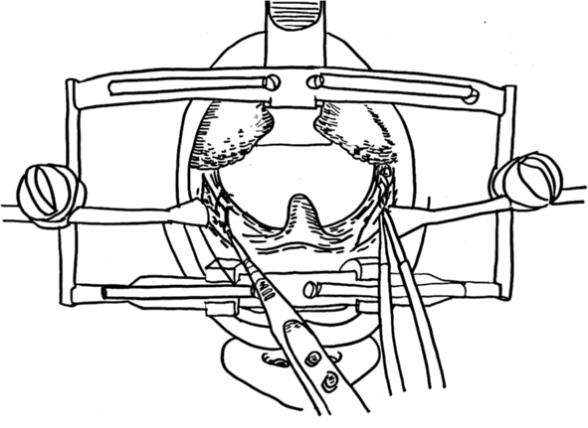
Meticulous hemostasis with monopolar and bipolar electrocautery before finishing the procedure

The surgeon preferably operates from the 12-o’clock position. Shoulder roll is placed and headrest is tilted for neck extension. After painting and draping, the retractor is placed for visualization. The Dingman retractor was used previously, but more complete visualization is achievable with the McIvor retractor. The senior author currently uses the McIvor retractors exclusively (Fig. 1). After the administration of a local anesthetic agent containing vasoconstrictor, medial portion of tonsil is grasped with curved or right-angled Kelly clamps (Fig. 2). While applying tension on the tonsil, the base of the tonsil is carefully dissected along the extratonsillar capsule until the tonsil is completely free from the base (Figs. 3 and 4). Dissection is mainly done with monopolar electrocautery with the aid of bipolar electrocautery for hemostasis as needed (Fig. 5). After the complete removal of the tonsils, the anterior and posterior tonsillar pillars are sutured together to further protect the surgical wound.

## Results

**Fig. 6:**
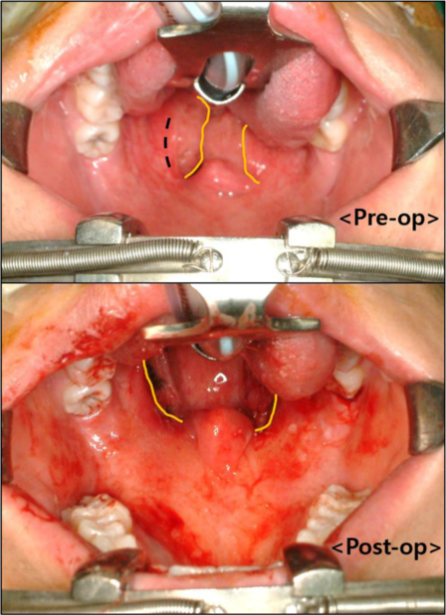
Pre-op and post-op clinical photos. *Dashed line*: location of incision and initial dissection. *Solid lines*: outline of airway before and after tonsillectomy

**Fig. 7:**
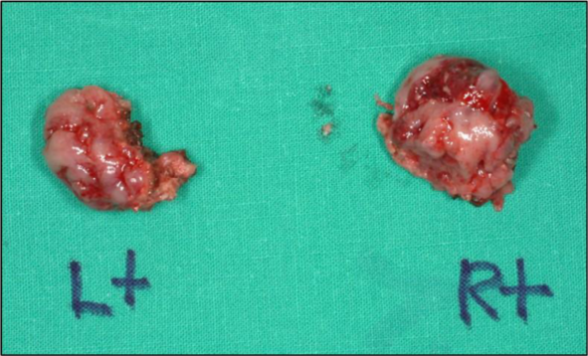
Removed tonsils

According to the chart review, 18 patients underwent tonsillectomy as part of the treatment of SDB, 2 patients underwent tonsillectomy for the treatment of SDB following mandibular setback surgery, 2 patients underwent tonsillectomy along with mandibular setback surgery in anticipation of postoperative airway obstruction, and 1 velopharyngeal insufficiency (VPI) patient received tonsillectomy along with posterior pharyngeal flap in anticipation of postoperative airway obstruction. Clinical photos of an example case are shown in Figs. 6 and 7.

The most common procedures performed along with tonsillectomy were uvulopalatopharyngoplasty (UPPP) and genioglossus advancement genioplasty which were done in 18 cases and 11 cases, respectively. Four patients received third molar extractions along with tonsillectomy. Other concomitant procedures included revision cheiloplasty (2), orthognathic surgery (2), plate removal (2), tongue base reduction (1), mass excision (1), zygoma augmentation (1), and posterior pharyngeal flap surgery (1).

The size of the tonsils was evaluated according to a grading scale suggested by Friedman in 2002. Tonsil grading varied from grade 1 (hidden within the tonsillar pillars) to grade 3 (tonsils beyond the pillars but not reaching midline). The majority of patients were diagnosed with grade 2 tonsillar hypertrophy (12 patients); 7 patients were grade 1, and 4 patients were grade 3.

Mainly concerned complications are hemorrhage, post-operative nausea and vomiting (PONV), respiratory complications, and postoperative pain. According to the chart review, none of the significant complications were noted (Table 1).

**Table 1:** Data from chart review

Number	Age at surgery	Sex	Diagnosis	Tonsils and soft palate status	Concomitant surgeries	Surgical outcome	Complications	Amount of mandibular setback	Notes
1	56	M	OSA	Hypertrophic	UPPP	Snoring improved	n/s	n/a	
2	24	F	OSA following mandibular setback	Hypertrophic	UPPP	Apnea, snoring improved	n/s	7.5 mm	OSA following mandibular setback
3	40	M	OSA, retrusive chin	Moderately enlarged, low soft palate	UPPP, genioglossus advancement (4 mm)	Apnea, snoring improved	n/s	n/a	
4	55	M	OSA, impacted third molars	Hypertrophic, low soft palate	UPPP, tongue base reduction (RF ablation), third molar extractions	Apnea, snoring improved	n/s	n/a	
5	28	M	OSA, impacted third molars	Hypertrophic	UPPP, third molar extractions	Snoring improved	n/s	n/a	
6	45	M	OSA, KCOT of ant. Maxilla	Hypertrophic	Mass excision, UPPP	Snoring improved	n/s	n/a	
7	28	F	OSA, impacted third molars	Hypertrophic	Third molar extractions	Snoring improved	n/s	n/a	
8	21	M	OSA, tonsillar hypertrophy, cleft lip nose deformity, cleft lip scar	Hypertrophic	Revision cheilorhinoplasty	Snoring improved	n/s	n/a	
9	24	M	OSA, retrusive chin	Hypertrophic, low soft palate	UPPP, genioglossus advancement (6 mm)	Apnea, snoring improved	n/s	n/a	
10	22	M	Mand. Prognathism, anterior open bite, malar depression, hypertrophic tonsils	Hypertrophic	Le Fort I osteotomy, BSSRO setback (Rt. 8 mm, Lt. 7 mm), zygoma augmentation with Medpor, Neurorrhaphy	No signs or symptoms of airway obstruction observed	IAN severance—>neurorhaphy	Rt. 8 mm, Lt. 7 mm	Preventive measure for post-op airway obstruction
11	44	M	OSA, retrusive chin	Moderately enlarged, low soft palate	UPPP, genioglossus advancement (4 mm)	Apnea, snoring improved	n/s	n/a	s/p ENT surgery at different hospital for OSA
12	8	M	VPI, hypertrophic tonsils, cleft lip scar	Hypertrophic	Posterior pharyngeal flap, revision cheiloplasty	No signs or symptoms of airway obstruction observed	n/s	n/a	Preventive measure for post-op airway obstruction
13	27	M	Mandibular prognathism, asymmetry of the jaws, tonsillar hypertrophy	Hypertrophic	Le Fort I osteotomy, BSSRO setback (Rt. 9.5 mm, Lt. 10.5 mm)	No signs or symptoms of airway obstruction observed	n/s	Rt. 9.5 mm, Lt. 10.5 mm	Preventive measure for post-op airway obstruction
14	32	M	OSA, retrusive chin	Hypertrophic, low soft palate	UPPP, genioglossus advancement (6 mm)	Snoring improved	n/s	n/a	
15	36	M	OSA, retrusive chin, retropositioned tongue base	Hypertrophic, low soft palate	UPPP, genioglossus advancement (4 mm)	Snoring improved	n/s	n/a	
16	44	M	Remaining snore after MMA	Hypertrophic	UPPP, plate removal	Snoring improved	n/s	n/a	
17	45	M	OSA	Hypertrophic, low soft palate	UPPP, genioglossus advancement (4 mm)	OSA, snoring improved	n/s	n/a	
18	63	M	OSA, retrusive chin	Hypertrophic	UPPP, genioglossus advancement (4 mm)	OSA, snoring improved	n/s	n/a	
19	25	M	OSA	Hypertrophic	UPPP, genioglossus advancement (4 mm)	OSA, snoring improved	n/s	n/a	
20	30	M	OSA, retrusive chin	Hypertrophic	UPPP, genioglossus advancement (4 mm)	OSA, snoring improved	n/s	n/a	
21	40	M	OSA, retrusive chin	Hypertrophic	UPPP, genioglossus advancement (4 mm)	OSA improved, snoring remains	n/s	n/a	
22	25	M	OSA, retrusive chin, impacted third molar (#38)	Hypertrophic	UPPP, genioglossus advancement (4 mm), third molar extraction (#38)	OSA, snoring improved	n/s	n/a	
23	22	M	OSA after mandibular setback	Hypertrophic	UPPP, plate removal	OSA, snoring improved	n/s	11 mm	OSA following mandibular setback

*n/a* not applicable, *n/s* none significant

## Discussion

Until the mid-1980s, the primary indication for tonsillectomy and adenotonsillectomy was recurrent throat infections. Beginning in late-1970s, Paradise and colleagues published a series of reports and randomized controlled trial results showing that only those children severely affected by throat infections benefitted from the removal of the tonsils, while moderate to minimally affected children merely showed modest benefit that may not outweigh the risk of surgery. These studies also revealed the self-limiting nature of throat infections [4–8]. Along with numerous studies questioning the efficacy of tonsillectomy, gradual decline in tonsillectomy rate was noted.

Currently recognized criteria warranting surgical removal of the tonsils are recurrent throat infections and SDB, with the latter being the more commonly found indication [1]. Recurrent throat infections indicative of tonsillectomy are defined as more than seven episodes of sore throats in 1 year, more than five episodes per year for 2 years, or more than three episodes per year for three consecutive years [9]. Each episode of sore throat should present with one or more of the following clinical signs or test results: temperature higher than 38.3 °C, cervical adenopathy, tonsillar exudates, or positive test for group A ß-hemolytic streptococci. If the frequency of sore throats is fewer than the above criteria, watchful waiting is recommended. However, if the patient has any of the modifying factors, surgical intervention is warranted. The modifying factors include multiple antibiotic allergies or intolerance, a combination of periodic fever, aphthous stomatitis, pharyngitis, and adenitis (PFAPA), or a history of peritonsillar abscess [9].

Sleep-disordered breathing (SDB) includes a broad range of signs, symptoms, and disorders from simple primary snoring at mildest to serious life-threatening disorders such as severe obstructive sleep apnea syndrome (OSAS) [10]. SDB is a disease of multifactorial cause at various levels of the upper airway [11]. Tonsillar hypertrophy is one of major contributing factors, and tonsillectomy has been shown to be beneficial in treating SDB in children with hypertrophic tonsils [12]. When only recurrent throat infections were considered, tonsillectomy was not a procedure commonly performed by oral and maxillofacial surgeons. With the shift in indication to pediatric and adult SDB, tonsillectomy has become a necessary tool for comprehensive care of SDB patients.

While all tonsillectomies in this report were done via conventional total extracapsular dissection method, partial intracapsular tonsillectomy is recently gaining attention for potentially lower complication rate and faster recovery. The main difference of intracapsular tonsillectomy from conventional total tonsillectomy is that a small portion of the tonsillar tissue along with the tonsillar capsule is left attached. It is theorized that this layer of attached tissue may prevent from damaging surrounding pharyngeal tissue, reducing post-operative discomfort and a chance of significant bleeding [13]. Recently, a microdebrider-assisted IT, also known as powered intracapsular tonsillectomy and adenoidectomy (PITA), has been shown to result in fewer post-operative complications and faster recovery [14]. The application of microdebrider is not limited to tonsillectomy and adenoidectomy but also include sinus surgery and nasal turbinectomy. The microdebrider seems to carry potential for various applications in the field of head and neck surgery.

The tonsillectomy procedure itself is not technically demanding, but unexpected excessive hemorrhage is a constant risk due to a surplus of blood supply to the tonsils and surrounding pharyngeal soft tissues. The superior tonsil pole is supplied by the descending palatine artery (DPA), the midfossa region by the ascending pharyngeal artery, and the inferior pole by the tonsillar and ascending palatine branches of the facial artery and the tonsillar branches of the lingual artery. In spite of the abundant circulation, no serious immediate or delayed postoperative hemorrhage was noted in this case series.

The main postoperative concerns are primary and secondary hemorrhage, postoperative nausea and vomiting (PONV), respiratory complications, and pain management. Primary hemorrhage (bleeding within the first 24 h of surgery) and secondary hemorrhage (bleeding more than 24 h after surgery, usually between 5 and 10 days) are reported to occur in 0.1 to 3 % of patients [15]. The best management strategy is meticulous intraoperative hemostasis using ligation, electrocautery, or coblation. However, for intraoperative or postoperative hemorrhages that cannot be controlled locally, external carotid artery (ECA) ligation is warranted in order to prevent life-threatening situations. Intraoperative administration of steroid (dexamethasone) has been shown to significantly reduce PONV [16]. Intraoperative intravenous steroid administration has also been shown to reduce postoperative pain [17]. Postoperative respiratory complications may result either from hemorrhage or edema. A clinical guideline from American Academy of Pediatrics recommends that children with cardiac complications of OSA, neuromuscular disorders, prematurity, obesity, failure to thrive, craniofacial anomalies, or a recent upper respiratory tract infection should be admitted overnight due to increased risk of postoperative respiratory complications [18].

Although not as thoroughly studied as pediatric population, tonsillectomy in the treatment of adult SDB patients has also been shown to be effective. Unlike the pediatric counterpart, the efficacy of tonsillectomy for the treatment of adult SDB lacks prospective randomized controlled trials and large-scale literature reviews. However, a number of retrospective studies report that, in carefully selected patients, tonsillectomy should be considered as one of the first surgical interventions for adult patients with SDB [19].

All patients included in this study were adults diagnosed with SDB or at high risk of developing airway stenosis due to mandibular setback surgeries. There have been reports of developing OSAS following mandibular setback surgery [20]. Also, the narrowing of pharyngeal airway space after mandibular setback has been studied and supported by a number of researches [21–23]. Therefore, when extensive mandibular setback surgery on patients with hypertrophic tonsils is planned, staged or concomitant tonsillectomy should be considered.

Since all tonsillectomies were performed along with other surgical treatment modalities such as UPPP, uvulopalatal flap, genioglossus advancement genioplasty, and orthognathic surgery, efficacy of tonsillectomy alone is difficult to assess. The most common concomitant procedure was UPPP. Since tonsillectomy is often required to precede UPPP, surgeons treating SDB patients should be capable of performing tonsillectomy in order to perform UPPP which is one of the most effective surgical procedures in treating SDB.

## Conclusions

SDB and OSAS are multifactorial disorders that are managed by various specialties such as pediatrics, otorhinolaryngology, neurology, oral medicine, orthodontics, sleep medicine, and oral and maxillofacial surgery. With established efficacy of tonsillectomy in treating childhood SDB and amassing evidence on the efficacy of tonsillectomy in treating adult SDB, tonsillectomy should be considered as a major tool among multifactorial armamentarium in treating OSAS and SDB. Since oral and maxillofacial surgeons are at the front line for surgical management of OSAS and SDB, oral and maxillofacial surgeons should stay updated on indications and surgical techniques of tonsillectomy and be capable of performing high-quality tonsillectomy on indicated patients.

## Abbreviations

DPA: Descending palatine artery
ECA: External carotid artery
OSAS: Obstructive sleep apnea syndrome
PFAPA: Periodic fever, aphthous stomatitis, pharyngitis, and adenitis
PITA: Powered intracapsular tonsillectomy and adenoidectomy
PONV: Post-operative nausea and vomit
QOL: Quality of life
SDB: Sleep-disordered breathing
UPPP: Uvulopalatopharyngoplasty
VPI: Velopharyngeal insufficiency
